# Die Leichenbücher des Wiener Anatomischen Instituts (1924–1959): Ein Fundstück und sein Wert als Quelle am Beispiel von neun Justifizierten in der Zweiten Republik

**DOI:** 10.1007/s00048-026-00440-0

**Published:** 2026-01-29

**Authors:** Leo Schaukal, Sophia Bauer

**Affiliations:** 1 Abteilung für Anatomie, Zentrum für Anatomie und Zellbiologie, Medizinische Universität Wien, Währinger Straße 13, 1090 Wien, Österreich; 2 Institut für Wirtschafts- und Sozialgeschichte, Universität Wien, Universitätsring 1, 1010 Wien, Österreich; 3 Doctoral School of Historical and Cultural Studies (DSHCS), Universität Wien, Wien, Österreich

**Keywords:** Anatomie, Wien, Zweite Republik, Leichenakquise, Todesstrafe, Anatomy, Vienna, Austria, Body Procurement, Death Penalty

## Abstract

In der Vergangenheit waren historische Forschungen zur Versorgung des Wiener Anatomischen Instituts mit Leichenmaterial in der Zwischenkriegszeit, während des Zweiten Weltkriegs und den ersten Jahren der Zweiten Republik dadurch erschwert, dass die Leichenbücher beziehungsweise Leicheneingangsbücher des Instituts (sie dienten der Dokumentation der persönlichen Daten Verstorbener) für diese Zeit als verschollen gegolten hatten. Stellte diese Lücke im Quellenbestand etwa bei den Forschungen zur NS-Zeit und dem Pernkopf-Atlas in den späten 1990ern und auch bisher ein großes Hindernis dar, so ermöglicht der dank eines Fundes (wieder) erschlossene Bestand sowohl dafür als auch für andere Forschungsbereiche neue, detaillierte Studien.

Der an der Wiener Abteilung für Anatomie archivierte Fund umfasst eine beinahe durchgehende Reihe der Wiener Leichenbücher von 1924 bis in die Zweite Republik. Dieser Beitrag verdeutlicht anhand eines kurzen Fallbeispiels – es widmet sich der Verbringung der Körper jener neun Personen, die während der Zweiten Republik von 1945 bis 1950 hingerichtet und im Anschluss an die Wiener Anatomie geliefert wurden –, die Aussagekraft und den Wert dieses lange verloren geglaubten und großen Quellenkorpus für neue historische Forschungsvorhaben sowohl als Einzelfallstudien als auch in Form serieller oder breit angelegter statistischer Untersuchungen.

## Abstract (en)

In the past, historical research concerning the body supply of the Viennese Anatomical Institute during the inter-war period, the Second World War and the early years of the Second Austrian Republic was largely hampered by the fact that the so called “Leichenbücher” or “Leicheneingangsbücher” (documenting every single body, body part, etc. the institute received) of the Anatomical Institute had been deemed lost. A recent find at the Department of Anatomy in Vienna has now provided us with this long missing source material reaching with a short interruption from 1924 all the way into the first decades of the Second Republic.

While this gap in source material has presented itself as an in some cases insurmountable issue for researchers, for example regarding the NS-era and the Pernkopf-Atlas during the late 1990ies, this find now provides ample opportunity for inquiries both for this time frame and beyond. This contribution shows the value the newly rediscovered books hold as source material based on a short study regarding the bodies of the nine individuals whose bodies were transferred to the Viennese Anatomical Institute after their execution during the Second Republic between 1945 and 1950. This example illustrates that this find will enable new research in the form of single case studies, serial inquiries and statistical analysis alike which without this discovery would remain impossible.

## Leichenbücher als Quellen für die Anatomiegeschichte im Nationalsozialismus und in der Nachkriegszeit

Im Sommer 2019 wurde im Zuge von Aufräumarbeiten in der Bibliothek der Abteilung für Anatomie des Zentrums für Anatomie und Zellbiologie der Medizinischen Universität Wien (vormals Institut für Anatomie der Universität Wien)^1^ ein Gutteil jener Leichenbücher aufgefunden, die in bisherigen Aufarbeitungen zur Anatomie in Wien zur Zeit des Nationalsozialismus und der Nachkriegsjahre als verloren gegolten haben. Die immense historische Bedeutung dieser Unterlagen, die dokumentieren, welche Leichen in einem bestimmten Zeitraum (dank des vorliegenden Fundes für Wien nunmehr fortlaufend ab 1924 mit einer einzigen Unterbrechung von 1940 bis 1942) an eine anatomische Einrichtung geliefert wurden, lässt sich anhand der rezenten Historiografie – im Speziellen zur nationalsozialistischen Anatomiegeschichte und darüber hinaus bis in die Nachkriegsjahre – erkennen.

Ab den 1980ern setzten sich akademische Einrichtungen im deutschsprachigen Raum (zum Beispiel die Universität Frankfurt oder das Anatomische Museum Hamburg) nach und nach mit der Frage auseinander, wie viele sterbliche Überreste von Opfern des NS-Staates sich zu dieser Zeit noch in den anatomischen und pathologischen Sammlungen des einstigen Dritten Reichs befanden (Weindling 2012: 238, 241). Seitdem existiert eine Reihe an Forschungsbeiträgen zur Geschichte der NS-Medizin, im Speziellen der NS-Anatomie, zur Frage der Nutzung der Körper von NS-Opfern durch anatomische Einrichtungen in allen Teilen des Dritten Reichs sowie der damit zusammenhängenden politischen beziehungsweise juristischen Verflechtungen.

Die Anatomie betreffend, bedienen sie sich dabei verschiedener Quellenarten – allen voran der Leichenbücher einzelner Institute, wenn sie vorhanden sind. Diese, so die Annahme, geben am umfassendsten beziehungsweise genauesten darüber Auskunft, welche Personen aus welchen Einrichtungen und zu welchem Zeitpunkt an die Anatomischen Institute gelangten und wie die Körper jener Verstorbenen innerhalb der Institute verwendet wurden. Jedoch betonen rezente Forschungsarbeiten, dass Leichenbücher als Quellengattung in Hinblick auf die Vollständigkeit ihrer Angaben beziehungsweise ihre Verlässlichkeit als aussagekräftige Quelle kritisch diskutiert und meist anderen Quellenarten gegenübergestellt beziehungsweise mit diesen vergleichend betrachtet werden müssen. Hier ist etwa *The Anatomy of Murder: Ethical Transgressions and Anatomical Science During the Third Reich* von Sabine Hildebrandt (Hildebrandt 2016) zu nennen, wo Leichenbücher als Untersuchungsmaterial unter anderem dahingehend problematisiert werden, als dass sie den Eingang der Leichen an Anatomische Institute nicht zwingend vollständig abbilden, da der Eingang mancher Körper teils auch gar nicht schriftlich erfasst wurde (Hildebrandt 2016, 187). Ob eine solche Ungenauigkeit im Falle Wiens vorliegt, gilt es in zukünftigen Untersuchungen zu klären.^2^

Leichenbücher sind als Quellen für die historische Aufarbeitung der Leichenversorgung der jeweiligen anatomischen Abteilungen des Dritten Reichs unerlässlich, wie die Beispiele Frankfurt und Wien zeigen. So verweisen Untersuchungen, die sich der Frage nach der Beschaffung der Anatomieleichen widmen, auf die wegen des Fehlens der Leichenbücher notwendig gewordene Diversität zu verwendender Quellen: In zwei aufeinander abgestimmten Artikeln die Lage in Frankfurt am Main betreffend substituieren Brehm et al. 2015 das vermutlich durch Bombentreffer zerstörte Leichenbuch I mit den für die Zeit verfügbaren Leichenpässen (Brehm et al. 2015a und Brehm et al. 2015b). Besonders relevant für den hier präsentierten Quellenfund ist das Fazit des Artikels, das lautete: „As is the case with other anatomical institutes […] we do not possess any regular body register for the years before 1944.“

Für Wien als weiteren Standort der NS-Anatomie und Fokus des Quellenfundes verweist der abschließende Bericht zum „Senatsprojekt der Universität Wien. Untersuchungen zur Anatomischen Wissenschaft in Wien 1938–1945“ aus dem Jahr 1998 (Akademischer Senat der Universität Wien 1998) ebenfalls auf den Bombentreffer am 10. Februar 1945, der – so die ursprüngliche Vermutung – den zeitgenössischen Bestand an Leichenbüchern vollständig zerstörte (Spann 1998: 184). Deshalb musste man sich, wie Brehm et al. (Brehm et al. 2015a und 2015b), für die Untersuchung, die unter anderem nach der Menge an jüdischen Opfern, aber auch politisch Verfolgten, die während des NS-Regimes an die Wiener Anatomie gelangten, fragte,^3^ anderer Quellen bedienen: normativer Dokumente, anderem Verwaltungsschriftgut und Zeitzeugenberichten (Mühlberger 1998: 29f.). Dieselben Limitationen ergaben sich etwa auch bei Untersuchungen hinsichtlich des für den sogenannten Pernkopf-Atlas (*Topographische Anatomie des Menschen, Atlas der regionär-stratigraphischen Präparation* [vier Bände: Pernkopf 1937, 1943, 1952 und 1957]) herangezogenen Leichenmaterials (jüngst etwa: Hildebrandt & Krebs 2025).

Neben Wien wurden bereits auch andere Standorte der NS-Anatomie im heutigen Österreich untersucht, für die jedoch im Vergleich zu Wien Leichenbücher vorhanden waren: Herwig Czech und Erich Brenner behandeln mit *Nazi victims on the dissection table – The Anatomical Institute in Innsbruck* (Czech & Brenner 2019) die Lage in Innsbruck, während es von Czech mit *Von der Richtstätte auf den Seziertisch. Zur anatomischen Verwertung von NS-Opfern in Wien, Innsbruck und Graz* (Czech 2015) eine gemeinsame Betrachtung der drei im heutigen Österreich gelegenen Anatomieinstitute des Dritten Reichs gibt. Der Schwerpunkt dieser Studie liegt dabei weniger auf Wien als auf Innsbruck und Graz. War für Innsbruck das Leichenbuch für den Zeitraum 1929 bis 1950 verfügbar und für Graz ebenfalls (allerdings fehlen hier präzise Angaben zu den Jahren, für die Leichenbücher zur Verfügung standen), stützte sich Czech in seiner Untersuchung für Wien auf die vom Fehlen der Leichenbücher geprägten Ergebnisse der Senatskommission.

Mittlerweile beschränkt sich die historische Aufarbeitung der Leichenbeschaffung im ehemaligen NS-Gebiet nicht mehr nur auf die NS-Zeit, sondern wurde ebenso auf die Zeit davor sowie auch auf die Nachkriegsjahre ausgeweitet: So beleuchtete Tatjana Buklijas konkret die Leichenversorgung des Anatomischen Instituts in Wien im Kontext etwa von interfakultärem Tauziehen, einer sich ändernden Stadt und wachsendem Antisemitismus als Gegenthese zum bis dahin den historischen Blick bestimmenden angelsächsischen Raum zwischen 1848 und 1914. Dabei musste ihre Untersuchung ohne Leichenbücher auskommen (Buklijas 2008: 575).

2018 versuchte sich Schütz an einem Gesamtüberblick des Leichenwesens Anatomischer Institute im deutschen Raum (vor allem Tübingen) mit Schwerpunkt auf dem Nationalsozialismus. Als Basis dafür lieferte er ebenfalls einen Abriss zum Thema der anatomischen Leichenversorgung im deutschsprachigen Raum, beginnend mit dem 18. und 19. Jahrhundert (Schütz 2018: 196–205). Anstelle von Leichenbüchern stand vor allem ein die Leichenbeschaffungspraxis anprangerndes Memorandum des Medizinstudenten Kurt Gerstein aus 1939 als Quelle im Zentrum dieser Studie.

Für Deutschland wurde weiters auch die Situation nach dem Zweiten Weltkrieg, insbesondere hinsichtlich der Versorgung anatomischer Einrichtungen mit Leichenmaterial, behandelt (Schütz et al. 2019, schwerpunktmäßig für München, oder Brehm et al. 2015a und 2015b bzw. Weiß et al. 2021 für die Dr. Senckenbergische Anatomie in Frankfurt am Main). Dazu stützte man sich unter anderem auf die zur Gänze erhaltenen Leichenbücher des Anatomischen Instituts München ab 1945 (Schütz et al. 2019: 109) und für die letzten Kriegsjahre beziehungsweise die Jahre nach dem Krieg auf das Leichenbuch II für Frankfurt am Main ab 1944 (Brehm et al. 2015a) beziehungsweise 1946 (Weiß et al. 2021).

Bisher keinen medizinhistorischen Beitrag gibt es im Gegensatz dazu zur Verbringung der Körper Hingerichteter an die anatomischen Institutionen Österreichs, speziell Wiens, während der Zweiten Republik, der sich dieser Beitrag im Rahmen einer kurzen exemplarischen Fallstudie widmet. Hier tut sich eine Forschungslücke auf, die den Verbleib der sterblichen Überreste all jener betrifft, die nach dem Recht der Zweiten Republik exekutiert wurden. Lediglich Martin Zellhofer hat diese Frage für einige konkrete Fälle aufgeworfen, die er in seiner Diplomarbeit *Die NS-Morde und -Standgerichtsfälle in Schwarzau im Gebirge und Umgebung im April/Mai 1945 im Lichte des Volksgerichtsverfahrens 1945–1948* behandelte. Sein Versuch, diese zu beantworten, war jedoch aufgrund des Mangels aussagekräftiger Primärquellen – vor allem der Leichenbücher – wenig erfolgreich (Zellhofer 2008: 138–140). Diese stehen nunmehr zu wesentlichen Teilen an der Wiener Abteilung für Anatomie als Primärquellen zur Verfügung und sollen in diesem Beitrag anhand einer Gruppe von neun Fallbeispielen kurz vorgestellt werden.

Die für die besagten Fallbeispiele der Justifizierten während der Zweiten Republik relevante Geschichte der Todesstrafe in Österreich während der Nachkriegsjahre ist dabei rechtshistorisch gut untersucht: Bernhard Sebl behandelt in seinem Beitrag „In Österreich zum Tode verurteilt“ die Geschichte der Todesstrafe in Österreich generell (Sebl 2008). Konkreter wird es bei Karl Haas in seinem Sammelbandbeitrag „Zur Frage der Todesstrafe in Österreich 1945 bis 1950“ (Haas 1995), der die historisch-politisch-juristische Basis für Hinrichtungen während der Zweiten Republik beleuchtet – etwa ihre verfassungsmäßige Stellung und legistische Implementierung. Für einen Teil der hier näher behandelten Fallbeispiele relevant ist *Volksgerichtsbarkeit und Verfolgung von nationalsozialistischen Gewaltverbrechen in Österreich 1945 bis 1972. Eine Dokumentation* von Karl Marschall (Marschall 1977), da vier der in den Wiener Leichenbüchern fassbaren Hingerichteten von Volksgerichten zum Tode verurteilt worden waren. Zum selben Gegenstand liegt von Claudia Kuretsidis-Haider eine aktuellere Studie vor, die „Todesurteile wegen NS-Verbrechen durch österreichische und alliierte Gerichte“ untersucht (Kuretsidis-Haider 2008).

## Wiederentdeckte Leichenbücher – Neues Forschungspotenzial

Wie auch an anderen deutschsprachigen Einrichtungen (Hildebrandt 2016: 187f.) dienten Leichenbücher schon vor der NS-Zeit der internen Dokumentation der Wiener Anatomischen Institute.^4^ Sie erfassen sämtliche eingelangte Körper Verstorbener mitsamt relevanten Informationen wie Name, Alter, Sterbedatum und -ort sowie Todesursache. Als historische Quellen sind diese Leichenbücher damit sowohl bei Fragestellungen, die Einzelfälle betreffen, als auch bei statistischen Erhebungen besonders aussagekräftig. Das Fehlen der Wiener Leichenbücher für den Zeitraum 1938 bis 1945 in den 1990er Jahren stellte folglich die Historikerkommission vor Probleme (Mühlberger 1998: 40), welche durch Heranziehung anderer Quellen nur bedingt kompensiert werden konnten. Dies beeinflusste die Ergebnisse ihrer Untersuchungen und damit den heutigen Forschungsstand merklich.

Schütz et al. erachten es für ihren Münchner NS-Forschungskontext als möglich, Leichenbücher, die im Zuge ihrer fortgesetzten Recherchen nur fragmentarisch von 1933 bis 1941 verfügbar waren (Schütz et al. 2017: 2), durch andere Quellen zu supplementieren (bereits in Schütz et al. 2013), wobei es sich hierbei um eine verbreitete Zugangsweise handelt (Brehm et al. 2015a und 2015b). Schütz et al. nutzten dazu für die 2013 publizierten Zwischenergebnisse zunächst im Speziellen Unterlagen aus dem Gefängnis Stadelheim, von dem die Anatomie München während der NS-Zeit die Körper Hingerichteter bezog, um basierend darauf etwa zu ermitteln, in welchem Umfang und in welcher Zusammensetzung nach Geschlecht oder Nationalität die Münchner Anatomie Körper Hingerichteter aus Stadelheim bezog. Für den Folgebeitrag von Schütz et al. aus dem Jahr 2017 fand eine nochmals erweiterte Palette an Quellen Eingang in die Untersuchung. Darunter, um nur einige Beispiele zu nennen, Daten aus dem Bundesarchiv Berlin-Lichterfelde (BAB), dem Bayerischen Hauptstaatsarchiv (BayHStA) oder dem Anatomischen Institut in Innsbruck (AII) (Schütz et al. 2017: 2). Basierend darauf war es möglich, die Rolle des Münchner Anatomischen Instituts innerhalb des NS-Systems von Hinrichtungsstätten und anatomischen Einrichtungen noch genauer zu erfassen – einerseits als vorrangiger unmittelbarer Profiteur, andererseits als zentrale Größe (Schütz et al. 2017: 9) bei der weiteren Verteilung der Körper Hingerichteter an andere anatomische Institute (Würzburg, Erlangen, Innsbruck).

2013 weisen auch Schütz et al. darauf hin, dass zum Beispiel noch keine Aussagen zur detaillierten Weiternutzung der Körper am Institut für Anatomie möglich sind (Schütz et al. 2013: 298–301). In ihrem Beitrag von 2017 konnte in diesem Kontext jedoch etwa aufgezeigt werden, dass für eine Dissertation aus dem Jahr 1944 sechs Körper hingerichteter Häftlinge herangezogen wurden (Schütz et al. 2017: 11), mitunter also durchaus detailliertere Ergebnisse zu erlangen sind. Ebenso liegt aus 2014 eine Untersuchung von Schütz et al. vor, die sich mit den Vitamin-C-Studien Max Claras, des Leiters der Münchner Anatomie ab 1942, befasst, der hierfür die Körper Hingerichteter als auch noch Hinzurichtender (seine Untersuchungen umfassten das Verabreichen von künstlicher Ascorbinsäure vor der Exekution) aus Stadelheim verwendete (Schütz et al. 2014).

2017 bestehen ungeachtet der erheblichen Forschungsleistung, die Schütz et al. über mehrere Jahre und Publikationen hinweg geleistet haben, weiterhin noch ein paar Limitationen: In gewissen Einzelfällen Hingerichteter sind weiterhin Details, etwa zur Verbringung der Körper bestimmter Personen, unklar (Schütz et al. 2017: 5). Und trotz der Vielzahl an herangezogenen Quellen ist es weiterhin nicht möglich, eine präzise Angabe zur Zahl der in München zwischen 1938 und 1945 zur Verfügung stehenden Körper zu machen. Lediglich Mindestangaben für einzelne zeitliche Abschnitte sind möglich (Schütz et al. 2017: 10f.). Anhaltende Unsicherheiten sind auch mit dem Fehlen der vollständigen Münchner Leichenbücher für diesen Zeitraum in Bezug zu setzen, was nach dem Krieg zu einer „almost unsurmountable confusion concerning the bodies’ [jenen aus Stadelheim, Anm. d. Verf.] whereabouts“ (Schütz et al. 2017: 11) führte.

Brehm et al. gehen ihrerseits für die Lage in Frankfurt am Main davon aus, mithilfe der anstelle des fehlenden Leichenbuchs herangezogenen Unterlagen ein nahezu vollständiges Bild der historischen Situation zeichnen zu können – auch für die Zeit, bevor Leichenbücher als Quellenmaterial ab 1944 wieder zur Verfügung stehen (Brehm et al. 2015a: 109). Insbesondere hinsichtlich der statistischen Aufarbeitung der verfügbaren Körper ist Brehm et al. trotz des Fehlens des korrelierenden Leichenbuchs eine dichte Rekonstruktion gelungen, vor allem dank des Vorhandenseins von Leichenpapieren^5^ für diese Zeitspanne. Limitationen ergaben sich primär durch fehlende Informationen bei einzelnen Aktenstücken oder bei ebenfalls herangezogenen Nachträgen im noch vorhandenen Leichenbuch II aus den Jahren 1942 bis 1944 sowie durch eine Lücke in den Leichenpapieren, die von Dezember 1938 bis Dezember 1940 reicht (Brehm et al. 2015a: 100). Die Kritik der Senatskommission zeugt für die Quellenlage in Wien jedoch davon, dass bestimmte Informationen weiterhin nur mithilfe von Leichenbüchern zu rekonstruieren sind, beziehungsweise davon, dass für Wien bisher kein anderes Quellenkorpus in ausreichendem Maße als Substitut verfügbar ist (Mühlberger 1998: 40). Breit angelegte Studien unter Einbeziehung diverser zusätzlicher Quellen aus dem In- und Ausland, wie sie etwa Schütz et al. speziell (2017) als Ergebnis jahrelanger Forschungsarbeit vorgelegt haben, stehen für Wien noch aus. Der Fund der Wiener Leichenbücher kann, so eine berechtigte Hoffnung, einen wesentlichen Beitrag dazu leisten, nicht nur derartige Untersuchungen zu ermöglichen, sondern womöglich auch erst anzustoßen.

Dank des Fundes im Jahr 2019 sind nunmehr fortlaufende Leichenbücher von 1924 bis zum 6. Jänner 1940 vorhanden, an dem die Eintragungen im betreffenden Leichenbuch enden. Das Fehlen des Bandes für den Folgezeitraum 1940 bis 1942 lässt sich am ehesten durch den bereits angesprochenen Bombentreffer erklären. Angesichts der Schilderung, die der Zeitzeuge Prof. Alfred Gisel (1911–2012)^6^ der Wiener Historikerkommission am 04.11.1997 in einem Interview gab, ist es gut vorstellbar, dass der nun fehlende Band sich gerade zur Bearbeitung im Sekretariat befand, als dieses durch den Bombentreffer im Februar 1945 zerstört wurde, während die übrigen im Keller gelagert waren, wo die Leichenanlieferung stattfand (Spann 1998: 184).

Ab 1942 besteht bis heute wieder eine durchgehende Reihe an Leichenbüchern, wenngleich rund um das Ende des Zweiten Weltkriegs entweder die Konsequenz bei den Eintragungen nachließ oder der Betrieb am Institut – infolge der Kriegswirren und des Bombentreffers Anfang 1945 (ebd. 1998: 177) – dermaßen eingeschränkt war, dass keine Anlieferung von Leichen mehr stattfand. Diese Schlussfolgerung ergibt sich aus einer Lücke bei den Eintragungen, die vom Jänner 1945 bis in den Oktober desselben Jahres reicht.

Die Einträge in den Leichenbüchern folgen dabei im Wesentlichen alle demselben Schema unter Angabe der weitgehend selben Eckdaten zu den einzelnen am Institut eingelangten Körpern Verstorbener: Name, Alter, Sterbedatum, Sterbeort (zum Beispiel Name des Krankenhauses oder „LG“ für „(Wiener) Landesgericht“), zuständiges Bestattungsunternehmen, Art der Leiche (zum Beispiel ganze Leiche, einzelne Körperteile, Gehirn, Fötus, etc.) und zusätzliche Anmerkungen – darunter etwa weitere Informationen zur Todesursache (zum Beispiel „justifiziert“ für hingerichtete Personen). Wie Sabine Hildebrandt in Bezug auf deutsche Universitäten zwischen 1933 und 1945 kritisiert, ist bei solchen Angaben allerdings dahingehend Vorsicht geboten, da zum Beispiel in Würzburg Euthanasie-Opfer oder in Marburg Zwangsarbeiter:innen^7^ in den Leichenbüchern nicht aufscheinen, also Unvollständigkeit gegeben sein kann. Andererseits, so Hildebrandt, sind auch Angaben über die Todesursachen mitunter zu hinterfragen, da Personen, die während der NS-Zeit zwar nicht hingerichtet wurden, aber anderweitig in Haft verstarben (z. B. aufgrund der schlechten Haftbedingungen), ebenfalls nicht unmittelbar in dieser Form gekennzeichnet wurden (Hildebrandt 2016: 187).

Die aufgefundenen Leichenbücher bieten unter Berücksichtigung dieser quellenkritischen Erwägungen die Möglichkeit, an die Arbeit der Wiener Historikerkommission anzuknüpfen. Darüber hinaus ermöglichen sie detailliertere Untersuchungen zur Wiener Anatomie sowohl der Zwischenkriegszeit als auch der Zweiten Republik. Die drei Phasen als zusammenhängende Abschnitte in der Geschichte der Wiener Anatomie verstehend, können die Leichenbücher ebenso einen zentralen Beitrag bei Untersuchungen leisten, die einen Bogen von der Ersten Republik und dem Austrofaschismus über den Nationalsozialismus bis in die Zweite Republik spannen – in Hinblick auf Beschaffung und Verwendung der Körper am Institut, demografische Aussagen zu den Verstorbenen oder das Verhältnis zwischen Anatomie und Strafvollzug. Auch stellen sie eine wichtige Quelle für transregionale Betrachtungen der Verhältnisse an anatomischen Einrichtungen sowohl während (beispielsweise Hildebrandt 2016: 187, 355–356) als auch nach dem Ende des Dritten Reichs dar.

Um dieses erweiterte Potenzial – auch hinsichtlich der Nachkriegszeit – zu veranschaulichen, folgen kurze Fallstudien, die aus den Leichenbüchern als Quellenbasis entwickelt wurden und deren Wert als Primärquellen illustrieren, da die folgende Thematik überhaupt erst durch sie behandelbar geworden ist, nämlich die Fälle von zum Tode verurteilten und auch tatsächlich hingerichteten Personen aus den ersten Jahren der Zweiten Republik, die in einem der Leichenbücher als „justifiziert“ ausgewiesen sind.

## Die Rechtslage in der Zweiten Republik

Einleitend ist eine knappe Betrachtung der juristischen Grundlagen der Todesstrafe und der gesetzlichen Regelungen bezüglich der Abgabe von Leichenmaterial an anatomische Einrichtungen in den Nachkriegsjahren basierend auf der eingangs vorgestellten Sekundärliteratur angebracht. Die juristische Basis hierfür ist lückenlos nachvollziehbar.

Mit Ausrufung der Zweiten Republik trat 1945 eine Verfassung in Kraft, in der „das Bundesverfassungsgesetz in der Fassung von 1929 wieder in Kraft gesetzt“ (Haas 1995: 398) wurde, womit die Todesstrafe abgeschafft war. Jedoch wurde dieses Verbot am 19. Juni 1946 zunächst bis 1947 ausgesetzt (sistiert)^8^ und die Sistierung durch den Nationalrat wiederholt bis 1950 verlängert.^9^ In der Folge wurden von 1945 bis 1950 einige Delikte nach dem Strafgesetz mit der Todesstrafe bedroht, wobei die meisten dieser Straftatbestände bereits im Zuge der austrofaschistischen Gesetzgebung erlassen wurden.^10^ Hinzu kam in den ersten Jahren der Zweiten Republik die Möglichkeit eines Todesurteils durch Volksgerichte (Garscha & Kuretsidis-Haider 1996: 2), die nationalsozialistische Verbrechen^11^ und Kriegsverbrechen^12^ verfolgten.

1950 wurde die Todesstrafe im österreichischen Strafrecht aufgehoben (Sabitzer 2010: 30), da die erneute Sistierung ihres Verbots im Nationalrat scheiterte (Haas 1995: 403). Die Todesstrafe blieb allerdings bis 1955 als Strafandrohung vor Volksgerichten und in der militärischen Standgerichtsbarkeit bis 1968 bestehen (Kuretsidis-Haider 2008: 85). Nach 1950 kam sie jedoch nie wieder zur Anwendung.

Was nun die für die Verbringung der Körper Hingerichteter vor und während der NS-Zeit relevanten rechtlichen Primärquellen und damit den Berührungspunkt der Todesstrafe mit dem Anatomischen Institut in Wien anbelangt, gibt Kurt Mühlberger in seinem Beitrag im Abschlussbericht des „Senatsprojekts der Universität Wien“ in wesentlichen Auszügen wieder: Ab 1928 existierte in Österreich eine Regelung für die Nutzung von Körpern von im Strafvollzug Verstorbenen als Studienleichen, was folglich alle Hingerichteten einschloss.^13^ Dies war in einem Erlass des Bundesministeriums für Justiz geregelt, der am 5. Juni 1928 veröffentlicht und am 16. April 1937 erneuert wurde: „Leichen von Strafgefangenen (ausgenommen Infektionsleichen) [sind], wenn sich bis zu dem Sterbetag folgenden zweitnächsten Tage 8 Uhr morgens keine Angehörigen finden, die für ein Leichenbegräbnis Sorge tragen und sich auch kein Angehöriger meldet, der gegen die Abgabe der Leiche zu Studienzwecken Einspruch erhebt, an das anatomische Institut in Wien, IX. Währingerstrasse 15 [sic!] zu übergeben.“^14^

Im Dritten Reich erging am 18. Februar 1939 ein Runderlass bezüglich der Abgabe der Leichen Hingerichteter an die anatomischen Abteilungen des Reichs im Kontext der „Neuregelung des Vollzugs der Todesstrafe“^15^: „Die Leichen der im Gebiete des Deutschen Reiches hingerichteten Personen sollen dem Anatomischen Institut der jeweils nächstgelegenen Universität zum Zwecke der wissenschaftlichen Forschung und des Unterrichts überlassen werden.“^16^ Es wurde daher bestimmt, „daß die Leichen Hingerichteter – soweit sie nicht von den Angehörigen in Anspruch genommen werden – […] von […] der Strafanstalt Wien den Anatomischen Instituten Wien und Graz zufallen.“^17^ Österreichisches und nationalsozialistisches Gesetz regelten die Abgabe der Körper Hingerichteter als Studienleichen also in weitgehend übereinstimmender Weise, wie die von Mühlberger zitierten Quellen belegen. Diese Regelungen dürften basierend auf dem „Rechts-Überleitungsgesetz“ vom 1.5.1945^18^ noch während der Zweiten Republik Bestand gehabt haben.

### Die Auswertung: Hingerichtete in den Leichenbüchern zu Beginn der Zweiten Republik

Vorbehaltlich möglicher den Nachkriegswirren geschuldeter Unvollständigkeiten in den Aufzeichnungen erlauben die aufgefundenen Leichenbücher konkrete Aussagen dazu, in welchem Umfang die Todesstrafe in den ersten Jahren der Zweiten Republik zur Versorgung der Wiener Anatomie mit Studienleichen beitrug. In Summe waren es neun Personen, die zwischen 1945 und 1950 nach österreichischem Recht hingerichtet, in dem betreffenden Leichenbuch verzeichnet und als Studienleichen an die Wiener Anatomie verbracht wurden: Josef Horvath (Körper eingelangt am Wiener Institut für Anatomie am 21.10.1947)^19^, Franz Mühlbacher (eingelangt am 30.04.1948)^20^, Johann Braun (eingelangt am 15.05.1948)^21^, Johann Wallner (eingelangt am 15.05.1948)^22^, Josef Wenninger (auch Weninger)^23^ (eingelangt am 15.05.1948)^24^, Josef Voggesberger (eingelangt am 30.09.1948)^25^, Karl Willmann (eingelangt am 09.04.1949)^26^, Josef Ostermayer (eingelangt am 01.06.1949)^27^ und Johann Trnka (eingelangt am 24.03.1950)^28^. Dass es sich hierbei ausschließlich um Männer handelt, ist dem Umstand geschuldet, dass alle in der Zweiten Republik gegen Frauen gefällten Todesurteile auf dem Gnadenweg in Kerkerstrafen umgewandelt wurden (Sebl 2008: 128).

Braun, Wenninger, Wallner und Voggesberger wurden wegen nationalsozialistischer Verbrechen beziehungsweise wegen Kriegsverbrechen von Volksgerichten zum Tode verurteilt. Der am 6. Dezember 1896 geborene (Marschall 1977: 117) Johann Braun, NS-„Kreisleiter“ von Neunkirchen, wurde am 24. Mai 1947 „wegen Verbrechen des vollbrachten gemeinen Mordes nach §§ 134, 135 Z 4 StG, wegen Verbrechen der Quälereien und Mißhandlung nach § 3 Abs. 1 und 2 KVG, wegen Verbrechen des Hochverrates nach § 58 StG in der Fassung der §§ 11, 10, VerbotsG. sowie wegen Kriegsverbrechens nach § 1 Abs. 2 und 6 KVG unter Bedachtnahme auf § 34 StG“ (ebd.: 118) zusammen mit dem HJ-Führer Johann Wallner und Josef Wenninger, Brauns einstigem „Stabschef“, vom Volksgericht Wien zum Tode verurteilt. Hinzu kamen in allen drei Fällen Verurteilungen auf Vermögensverfall. Allen dreien wurde zur Last gelegt, „im Mai 1945 in Schwarzau am Gebirge ein ‚Standgericht‘ gebildet“ zu haben. Ein Mitangeklagter wurde zu lebenslänglichem Kerker verurteilt, ein weiterer freigesprochen.

Gnadengesuche und Anträge auf Wiederaufnahme der Verfahren durch Braun, Wallner und Wenninger beziehungsweise ihre jeweiligen Anwälte – teils unter Mitwirkung von Angehörigen – wurden abgelehnt. Alle drei zum Tode Verurteilten wurden in unmittelbarer zeitlicher Nähe am 15. Mai 1948 hingerichtet. Das Gerichtsurteil legte auch die dabei einzuhaltende Reihenfolge der Hinrichtungen fest: Als erste erfolgte jene von Wenninger, dann die von Wallner und zuletzt die Brauns. Der ursprünglich vorgesehene Hinrichtungstermin am 12. Mai 1948, von dem die Verurteilten nur einen Tag zuvor in Kenntnis gesetzt worden waren, war aufgrund kurzfristig eingebrachter Anträge auf Wiederaufnahme der Verfahren nicht eingehalten worden. Die Hinrichtungen erfolgten ab 6 Uhr morgens im Beisein einer Gerichtskommission – darunter Richter, Staatsanwalt und Gerichtsarzt. Auch die Anwälte der Verurteilten waren zugegen. Beim ersten Hinzurichtenden, Josef Wenninger, stellte der Gerichtsarzt um 6:30 Uhr den Tod fest, bei Johann Wallner um 6:35 Uhr und bei Johann Braun um 6:41 Uhr. Das Protokoll vermeldet abschließend schlicht: „Hiermit ist der angeordnete Strafvollzug ausgeführt und beendet.“^29^

Noch am selben Tag, dem 15. Mai 1948, trafen die drei Körper am Institut für Anatomie der Universität Wien ein. Hier wurden sie entsprechend der österreichischen Methode der Hinrichtung durch den Würgegalgen^30^ (Sabitzer 2010: 31) als „Ganze Leichen“ (andere Kategorien waren etwa „Kinderleiche“, „Fötus“, „Sezierte Leiche“ oder „Gehirne“) unter Angabe ihres Alters (Johann Braun 52 Jahre, Johann Wallner 29 Jahre und Josef Wenninger 49 Jahre), des Datums, ihres Namens, Sterbetags, Sterbeortes (LG für Landesgericht) und der zuständigen Bestattung (GW für Gemeinde Wien) verzeichnet. Diesem Schema folgend wurden sämtliche Eingänge von Studienleichen mit ihren Eckdaten erfasst. Bei allen dreien wurde festgehalten, dass sie „justifiziert“ worden waren. Martin Zellhofer hatte über den Verbleib der Körper von Braun, Wenninger und Wallner in seiner Diplomarbeit basierend auf Auskünften der Wiener Magistratsabteilung 43 (Wiener Friedhöfe) und den Friedhöfen in den Heimatorten der Hingerichteten, die keinerlei passende Unterlagen ausfindig machen konnten, nur mutmaßen können: „Die sterblichen Überreste der Verurteilten wurden entweder in ihre Heimat überführt oder in Gräbern ohne namentliche Erfassung der bestatteten Personen begraben.“ (Zellhofer 2008: 138–140) Beide von Zellhofer in Betracht gezogenen Erklärungen sind dank der Wiener Leichenbücher somit unzweifelhaft als unzutreffend einzustufen. Die Körper von Braun, Wenninger und Wallner wurden auf keinem Friedhof beigesetzt, sondern gelangten nach ihrer Exekution an die Wiener Anatomie.

Der deutsche Staatsbürger Josef Voggesberger wurde am 24. April 1948 durch das Volksgericht Wien zum Tode verurteilt. Der Schuldspruch erfolgte wegen „Mord, Quälerei und Mißhandlung, Verletzung der Menschlichkeit und Menschenwürde als KZ-Aufseher in Dachau“ (Butterweck o.J.). Die Staatsanwaltschaft Wien befand sich als zuständig, da Voggesberger „an den Misshandlungen von KZ.-Häftlingen, insbesondere auch von österreichischen Häftlingen beteiligt war.“ Eine Auslieferung „an Deutschland wegen des [sic!] von ihm in Dachau an nichtösterreichischen Staatsbürgern begangenen Verbrechen“ kam für das österreichische Justizministerium 1948 nicht infrage, „da in Deutschland keine souveräne Regierung besteht, welcher die Auslieferung des Genannten [also Voggesbergers; Anm. d. Verf.] angeboten werden könnte.“^31^ Seine Hinrichtung erfolgte am 30. September 1948 und stellt damit das letzte Todesurteil dar, das von einem österreichischen Volksgericht ausgesprochen und tatsächlich vollstreckt wurde (Kuretsidis-Haider 2008: 97), nachdem mehrere Gnadengesuche von Voggesberger und seiner Ehefrau abgelehnt worden waren.

Diese Ablehnung von Gnadengesuchen, wie auch in den Fällen von Braun, Wenninger und Wallner geschehen, passt zur statistischen Tendenz bei Todesurteilen vor Volksgerichten. Dort hatten Gnadengesuche viel geringere Erfolgsaussichten als bei Todesurteilen vor Strafgerichten. Gleichwohl wurden andere, teils später erfolgte volksgerichtliche Todesurteile durch Begnadigungen in Kerkerstrafen umgewandelt. Voggesbergers Leichnam wurde noch am Tag der Exekution an die Wiener Anatomie überstellt und im Leichenbuch verzeichnet. Die dabei aufgenommenen Daten stimmen mit jenen überein, die sich bei anderen finden, ebenfalls der Vermerk „justifiziert“. Josef Voggesbergers Alter wird mit 40 Jahren angegeben.

Die übrigen Hingerichteten wurden auf Basis von Urteilen in regulären Strafprozessen justifiziert: Josef Horvath, zum Zeitpunkt seiner Hinrichtung 23 Jahre alt, wurde am 21. Oktober 1947 im Wiener Landesgericht hingerichtet. Seine Verurteilung zum Tode erfolgte im August 1947 wegen des Mordes an einer 66 Jahre alten Frau, mit der Horvath eine Beziehung geführt und die er im Zug eines Streits im Juni 1946 im Burgenland erschlagen hatte. Seine sterblichen Überreste trafen noch am 21. Oktober 1947 in der Wiener Anatomie ein. Bei Horvath wurde als zusätzlicher Vermerk „hingerichtet“ angegeben.

Der zum Zeitpunkt seiner Hinrichtung 24-jährige Franz Mühlbacher wurde am 30. April 1948 im Wiener Landesgericht justifiziert. Er war am 18. Juni 1947 vom Kreisgericht St. Pölten wegen Doppelmordes zum Tode verurteilt worden, nachdem wegen „besonderer Grausamkeit und Kaltblütigkeit“ der Mordtat alle Berufungsanträge und im März 1948 auch der Antrag auf Wiederaufnahme des Verfahrens mangels neuer Beweislage abgewiesen worden waren. Auf das Stellen eines Gnadengesuchs wurde gänzlich verzichtet, wie die betreffende Gerichtsakte erkennen lässt.^32^ Im Leichenbuch der Wiener Anatomie wurde der Eingang seines Körpers noch am 30. April 1948 verzeichnet.

Karl Willmann, Schmiedegehilfe aus Hagenberg im Bezirk Mistelbach, wurde am 19. November 1948 wegen des Mordes an seiner 42-jährigen Ehefrau, seiner vierjährigen Tochter und seiner 70-jährigen Schwiegermutter zum Tode verurteilt. Die Vollstreckung des Urteils erfolgte am 8. April 1949 frühmorgens im Wiener Landesgericht. Sein Körper wurde am Folgetag, dem 9. April 1949, zur Nutzung als Studienleiche an das Wiener Institut für Anatomie überstellt. Dies zeigt ein Eintrag im Leichenbuch des Instituts, der mit dem bereits bekannten Schema übereinstimmt, Willmanns Alter mit 38 Jahren angibt und ihn auch als justifiziert kennzeichnet.

Josef Ostermayers Leichnam traf am 1. Juni 1949 auf der Anatomie Wien ein. Dem war ein gewisses Hin und Her vorangegangen. Ostermayer war bereits zwei Mal in separaten Verfahren wegen dreifachen Raubmordes zum Tode verurteilt worden, da er kurz vor dem ursprünglichen Hinrichtungstermin angegeben hatte, neue Beweise seiner Unschuld erbringen zu können, was zu einer Neuaufnahme des Verfahrens geführt hatte. Auch dieser erneute Prozess hatte jedoch mit der Verurteilung zum Tode geendet. Als Termin der auf dem zweiten Schuldspruch basierenden Exekution war der frühe Morgen des 1. Juni 1949 angesetzt. Auch diese Hinrichtung wurde im letzten Moment noch einmal aufgeschoben, da Ostermayer um Wiederaufnahme seines Verfahrens ersuchte. In der Folge verkündete das Landesgericht, bis um 14 Uhr des 1. Juni 1949 weitere Angaben in der Sache zu machen. Der Richtersenat des OGH bewertete Ostermayers Wiederaufnahmeantrag noch am selben Tag abschlägig. Die Hinrichtung erfolgte ebenfalls am selben Tag um 16 Uhr im Landesgericht Wien. Im Anschluss wurde die Leiche an das Anatomische Institut überführt und hier im Leichenbuch verzeichnet. Ostermayers Alter wurde mit 24 Jahren angegeben. Auch bei ihm entsprechen die sonstigen Angaben dem bekannten Schema, inklusive des Vermerks „justifiziert“. In diesem Fall wurde zusätzlich die weitere Verwendung der Studienleiche als „NÖ maz.“ verzeichnet. Wie die Zusammenschau mit einem Vermerk an anderer Stelle nahelegt, meint dies wohl „Nöbauer mazeriert“ und ermöglicht eine Nachvollziehbarkeit der präzisen weiteren Nutzung einer Studienleiche in der zweiten Hälfte der 1940er Jahre. Aus dem Leichnam Ostermayers wurde also ein Skelett angefertigt, – in diesem Fall durch Johann Nöbauer, einen Mitarbeiter des Instituts. Möglicherweise wurde bei den meisten eingelangten Leichen (egal, ob es sich um justifizierte oder andere Personen handelte) auf einen Verweis zur weiteren Verwendung verzichtet, wenn sie im üblichen Standardprozedere zur Nutzung als Studienmaterial im Rahmen der Sezierkurse perfundiert wurden. Darauf deutet hin, dass dieses Prozedere nirgends explizit vermerkt wurde, obwohl es zweifelsohne routinemäßig stattfand, während sich Vermerke über andere Formen der Weiternutzung (zum Beispiel „maceriert für Museum“ oder „Kopf Dr. Allmer“) beziehungsweise in anderen Fällen die Nicht-Weiternutzung (zum Beispiel „unbrauchbar“ oder „Leiche abgeholt“) regelmäßig in den Leichenbüchern finden.

Der 37 Jahre alte Johann Trnka war die letzte Person, die in der Zweiten Republik zum Tode verurteilt und hingerichtet wurde (Sabitzer 2010: 70). Da sein Körper der Wiener Anatomie übergeben wurde, handelt es sich zugleich um den letzten Fall, in dem die Leiche eines Justifizierten an ein österreichisches anatomisches Institut kam. Johann Trnka wurde in einem vom 12. bis zum 14. Dezember 1949 dauernden Gerichtsprozess am Wiener Straflandesgericht wegen des „zweifachen heimtückischen Raubmordes und siebenfachen versuchten Raubmordes sowie wegen Veruntreuungen zum Tod durch den Strang verurteilt“ (Sabitzer 2010: 71). Ein Gnadengesuch lehnte der Bundespräsident ab, sodass die Exekution am 24. März 1950 stattfand. Zwei Monate später beschloss der Nationalrat, die Sistierung des verfassungsmäßigen Verbots der Todesstrafe im ordentlichen Strafprozess nicht erneut zu verlängern, was Johann Trnka folglich zum letzten Hingerichteten der Zweiten Republik machte. Sein Leichnam wurde noch am 24. März 1950 an das Wiener Institut für Anatomie überstellt. Das Leichenbuch verzeichnet die üblichen Informationen mit dem Vermerk „justifiziert“. Trnka findet sich darüber hinaus in einem weiteren Aktenbestand des Wiener Anatomischen Instituts, nämlich in den Unterlagen, die als „Leichenbegleitscheine 1949–1952“ gekennzeichnet sind, jedoch auch verschiedenste andere Dokumente beinhalten, die im Zusammenhang mit der Leichenversorgung stehen – so in einem Schreiben, das am 16. Mai 1950 vom Anatomischen Institut an die Verwaltung des Wiener Zentralfriedhofs erging. In diesem Schreiben meldet das Institut die jüngst erhaltenen Studienleichen an die Verwaltung des Zentralfriedhofs, darunter unter „Eingelangt am 24.III.1950“ „Trnka, Johann, geb. 21.03.1912, gest. 24.III.1950, Landesgericht für Strafsachen“^33^.

Dass andere hier behandelte Justifizierte in derartigen an die Zentralfriedhofsverwaltung gerichteten Schreiben aufscheinen, ist jedoch ausgeschlossen: Die Vereinbarung, laut welcher das Anatomische Institut dem Zentralfriedhof so Bericht über eingegangene Studienleichen erstattet, trat erst per 12. Dezember 1949 und damit nach allen anderen acht Hinrichtungen in Kraft, wie ein vom Magistrat der Stadt Wien (MA 43, Friedhöfe) verfasstes Schreiben aus den Beständen des Archivs des Anatomischen Instituts zeigt.

Aus den Leichenbüchern gehen zudem nähere Details zur Überstellung an das Wiener Anatomische Institut hervor, allerdings nur in Kombination mit zusätzlichen Quellen – beispielsweise mit zeitgenössischen Zeitungsartikeln, den Todesbescheinigungen^34^ und den Gerichtsakten des Wiener Straflandesgerichts^35^. So erfolgte (üblicherweise) noch am selben Tag der meist frühmorgendlichen Hinrichtung^36^ die Anlieferung der Körper vom Wiener Straflandesgericht in der Landesgerichtsstraße (im 8. Wiener Gemeindebezirk) an das Anatomische Institut, das sich nicht weit entfernt in der Währinger Straße (im 9. Wiener Gemeindebezirk) befand und noch heute befindet. Weiters wird klar, dass die Wiener Anatomie den Körper des letzten Hingerichteten der Zweiten Republik, des Raubmörders Johann Trnka,^37^ erhielt.^38^ Worüber die Leichenbücher lediglich in Ausnahmefällen – unter den Hingerichteten etwa nur bei Josef Ostermayer –39 explizit Auskunft geben, ist die weitere Verwendung angelieferter Körper innerhalb des Instituts für Anatomie.

Die zahlenmäßige Einordnung der Fälle, in denen Körper Hingerichteter während der Zweiten Republik an die Wiener Anatomie kamen, zeigt in Bezug auf die Gesamtzahl der Hingerichteten: Bei insgesamt 46 in der Zweiten Republik erfolgten Hinrichtungen^40^ stellen die neun Justifizierten rund ein Fünftel und somit einen signifikanten Anteil aller Hingerichteten dar. Zwar erlaubt dies keinen allgemeinen Schluss über die Verbringung der sterblichen Überreste Hingerichteter während der Zweiten Republik, doch lässt sich zweifelsfrei belegen, dass es sich bei der Abgabe der Körper der neun Justifizierten an die Wiener Anatomie nicht etwa um ein Kuriosum handelte.

Gleichwohl erhielt die Wiener Anatomie nicht alle Körper, die in Wien durch Hinrichtungen anfielen. Geht man nämlich davon aus, dass alle Personen, die vor Wiener Gerichten zum Tode verurteilt und nicht begnadigt wurden, auch in Wien hingerichtet wurden – eine gerechtfertigte Annahme, da, wie das Beispiel Mühlbacher zeigt, sogar Hinrichtungen andernorts Verurteilter (St. Pölten) in Wien stattfinden konnten –, so zeigt ein Abgleich der durch das Volksgericht Wien zum Tode verurteilten Personen mit dem entsprechenden Leichenbuch des Anatomischen Instituts, dass viele in Wien Justifizierte dort nicht aufscheinen.^41^ Weiters ist zu bemerken, dass keine in anderen Justizanstalten der Zweiten Republik (zum Beispiel am Landesgericht Linz) Hingerichteten am Wiener Anatomischen Institut nachweisbar sind.

In welcher Proportion stehen die neun Körper Justifizierter, die im Zeitraum von 1945 bis 1950 an die Wiener Anatomie gelangten, zur Gesamtsumme der dem Institut als Studienleichen in diesem Zeitraum zur Verfügung stehenden Körper? Eine quantitative Auswertung des Leichenbuchs von Mitte Oktober 1945 (Start der Eintragungen nach Ende des Zweiten Weltkriegs) bis 1. Juli 1950, dem Ende der Todesstrafe in Österreich, ergibt insgesamt 314 an der Wiener Anatomie eingelangte „Ganze Leichen“.^42^ Selbst in den „Mangeljahren“ der frühen Zweiten Republik sind die neun Körper von Hingerichteten, die in diesen Zeitraum fielen, gegenüber der Gesamtmenge an „Ganzen Leichen“ vernachlässigbar (2,87 %).

### Ein neues Quellenkorpus für zukünftige Forschung

Das behandelte Fallbeispiel zeigt, wie groß der Wert der an der Wiener Anatomie verfügbaren Leichenbücher als zusätzliche Quellen ist. In der Zusammenschau mit bereits vorliegenden Forschungsergebnissen und anderen Primärquellen erlauben sie es, bisher offen gebliebene Forschungsfragen und gänzlich neue Forschungsgebiete – wie das hier knapp dargestellte Themenfeld der Nutzung der Körper Hingerichteter in der Wiener anatomischen Forschung und Lehre während der ersten Jahre der Zweiten Republik – zu behandeln.

Die Frühzeit der Zweiten Republik stellt jedoch nur einen Bruchteil der durch die vorliegende Untersuchung um zusätzliche wertvolle Quellen bereicherten Phase dar. Ähnlich fruchtbringende Erkenntnisse sind auch für die 1920er und 1930er Jahre sowie für die NS-Zeit zu erwarten – etwa durch eine Vertiefung der Ergebnisse der Senatskommission und der übrigen angeführten Forschung. Gleichzeitig ermöglichen sie, wenngleich stets unter Vorbehalt der potenziellen Unvollständigkeit von Angaben, die Behandlung einer Bandbreite an Themen und Fragestellungen: Sowohl Einzelfallstudien als auch serielle und statistische Erhebungen (zum Beispiel zu den Todesursachen) sind möglich. Auch drängt sich der Vergleich zwischen verschiedenen Standorten der Anatomie im deutschsprachigen Raum oder dem ehemaligen NS-Gebiet auf. Beispielsweise wäre ein Vergleich mit München, basierend auf den bereits vorliegenden Untersuchungen von Schütz et al. (Schütz et al. 2013, 2017 und 2019), oder mit Frankfurt am Main, behandelt durch Weiß et al. für die Nachkriegszeit (Weiß et al. 2021) und Brehm et al. (Brehm et al. 2015a und 2015b) gut vorstellbar. Dabei wäre etwa die Frage nach der Nutzung von Leichen Hingerichteter zu untersuchen und ob es diesbezüglich einen Unterschied zwischen Deutschland und Österreich gab. Allein die Ergebnisse dieser knapp angelegten Studie zeigen deutlich das Potenzial, das den Leichenbüchern als neu verfügbare Quellen innewohnt. Sie erwecken die berechtigte Hoffnung auf zahlreiche und inhaltlich vielfältige neue Forschungsvorhaben.

## Footnotes

1 Seit der Gründung der Medizinischen Universität Wien im Jahr 2004 besteht das vormalige Institut für Anatomie als Abteilung für Anatomie der Medizinischen Universität Wien als Teil des Zentrums für Anatomie und Zellbiologie fort.

2 Hildebrandts konkrete quellenkritische Anmerkung, wonach in Fällen aus Deutschland etwa Personen, die während der NS-Zeit aufgrund der schlechten Haftbedingungen verstarben, nicht ohne Weiteres als Opfer der NS-Justiz in den betreffenden Leichenbüchern erkennbar sind, dürfte für Wien nicht in dieser Form zutreffen. Zwar wären diese naturgemäß nicht als „justifiziert“ oder „hingerichtet“ ausgewiesen, jedoch würde der vermerkte Todesort (etwa LG für Landesgericht) einen klaren Hinweis darauf geben, dass es sich um im Strafvollzug Verstorbene handelt. In Kombination mit der angegebenen Todesursache besteht also für Wien in solchen Fällen eine gut nachvollziehbare Quellenlage.

3 Die Historikerkommission benennt die Zahl der Opfer der NS-Justiz, die zwischen 1938 und 1945 an das Wiener Anatomische Institut geliefert wurden, mit 1377 Personen. Mit Ausnahme von fünf zum Tode Verurteilten, die noch vor dem Exekutionstermin krankheitsbedingt verstarben, erfolgten alle hiervon betroffenen Hinrichtungen entweder am Wiener Landesgericht oder der Schießstätte Wien Kagran (Angetter 1998: 86).

4 Beziehungsweise früher dem Institut mit seinen historisch zwei bis vier separaten Lehrkanzeln.

5 In Wien: „Leichenbegleitscheine“. Diese sind für Wien laut aktuellem Kenntnisstand erst für die Zeit nach dem Ende des Zweiten Weltkriegs vorhanden.

6 Gisel war von 1973 bis 1981 Professor für Anatomie in Wien. Zuvor war er zwischen 1933 und 1944 bereits als Demonstrator und Assistent am Wiener Anatomischen Institut aktiv sowie erneut von 1947 bis 1948. Ab 1951, nach seiner Habilitation, arbeitete er wiederum in Wien als Universitätsdozent. Folglich kannte er Pernkopf und das Wiener Anatomische Institut vor, während und nach der Nazi-Zeit aus persönlicher Anschauung.

7 Aus Hildebrandts Formulierung im Englischen lässt sich leider nicht eruieren, ob es sich tatsächlich um Männer und Frauen gehandelt hat.

8 BGBl. Nr. 141/1946, § 1.

9 „Ausgewählte Artikel aus dem österreichischen Bundes-Verfassungsgesetz, Artikel 85“, online unter: http://www.nachkriegsjustiz.at/service/gesetze/gs_b-vg.php (25.06.2025).

10 BGBl. Nr. 77/1934.

11 StGBl. Nr. 13/1945, Art. 5, § 24.

12 StGBl. Nr. 32/1945, § 13.

13 Die Nutzung der Körper von in der Strafjustiz verstorbenen Personen ist im Kaiserreich Österreich-Ungarn bereits für die Zeit zwischen 1848 und 1914 belegt (Buklijas 2008: 4). Darüber hinaus war dies auch in deutschen Gebieten eine gängige Praxis (Schütz 2018: 196).

14 OeStA (Österreichisches Staatsarchiv), AVA (Allgemeines Verwaltungsarchiv) BMU Signatur 4G Fasz. 810, 12674-I/2/1928, zit. n. Mühlberger (1998: 43).

15 MedF 1131 aus 1938/39 und AVA BMU Signatur 4G Fasz. 810, GZ 311490-2d/39, zit. n. Mühlberger (1998: 45).

16 Ebd.

17 Ebd.

18 StGBl. Nr. 6/1945, §§ 1 und 2.

19 AIA (Archiv des Anatomischen Institutes Wien), Leichenverwaltung und Körperspende, Leichenbücher 1945–2016, LB 1945–1959.

20 Ebd.

21 Ebd.

22 Ebd.

23 „Todesurteile österreichischen [sic!] Volksgerichte 1945 bis 1948“, online unter: http://www.nachkriegsjustiz.at/prozesse/volksg/Tabelle_Todesurteile.php (25.06.2025).

24 AIA, Leichenverwaltung und Körperspende, Leichenbücher 1945–2016, LB 1945–1959.

25 Ebd.

26 Ebd.

27 Ebd.

28 Ebd.

29 Protokoll v. 15./5. [sic!]1948 über den Vollzug der Todesstrafe an 1.) Josef Weninger, 2.) Johann Wallner und 3.) Johann Braun, WStLA (Wiener Stadt- und Landesarchiv), LG Wien Vg 1b Vr 1693/45.

30 Der Würgegalgen wurde in Österreich ab 1873 für Hinrichtungen verwendet. Unter der NS-Herrschaft wurde diese Praxis durch die deutsche Methode der Enthauptung durch das Fallbeil (am Wiener Landesgericht: „Gerät F“) ersetzt (Sabitzer 2010: 31). Seine intensive Anwendung war der Grund dafür, dass die Wiener Anatomie während der NS-Zeit die Körper von mindestens 1377 Hingerichteten erhielt (Czech 2015: 144), womit sie wie andere anatomische Einrichtungen des Dritten Reichs gleichzeitig als integraler Bestandteil des NS-Hinrichtungswesens fungierte (Schütz 2018: 205).

31 Schreiben des BMJ (Bundesministerium für Justiz) an das Landesgericht für Strafsachen Wien vom 16.01.1948, WStLA, Volksgericht, A1 – Vg Vr-Strafakten: Vg 11 Vr 8456/46.

32 NÖLA (Niederösterreichisches Landesarchiv), KG St. Pölten VR 2001/46.

33 AIA, Leichenverwaltung und Körperspende, Leichenbegleitscheine, LBS 1949–1952, Schreiben des Anatomischen Instituts an die Verwaltung des Zentralfriedhofs, Auskunft vom 16.05.1950.

34 WStLA (Wiener Stadt- und Landesarchiv), Abt. 212, A 12 – Todesbescheinigungen: Totenbeschaubefunde für Karl Willmann, Johann Braun, Josef Wenninger, Josef Ostermayer, Johann Trnka, Josef Voggesberger und Franz Mühlbacher.

35 Siehe bspw. Protokoll v. 15./5. [sic!] 1948 über den Vollzug der Todesstrafe an 1.) Josef Weninger [sic!], 2.) Johann Wallner und 3.) Johann Braun, WStLA, LG Wien Vg 1b Vr 1693/45.

36 Anonym 1949. Der Schmied von Hagenberg heute früh hingerichtet. *Weltpresse* 83, Abendausgabe: 1.

37 Anonym 1949. Todesstrafe für Frauenmörder. Volkswille 291: 3.

38 AIA, Leichenverwaltung und Körperspende, Leichenbücher 1945–2016, LB 1945–1959.

39 AIA, Leichenverwaltung und Körperspende, Leichenbücher 1945–2016, LB 1945–1959: Josef Ostermayers sterbliche Überreste wurden mazeriert – also zu Knochenpräparaten verarbeitet.

40 Insgesamt 100 Todesurteile wurden in der Zweiten Republik gefällt, mit 46 jedoch weniger als die Hälfte davon auch vollstreckt.

41 AIA, Leichenverwaltung und Körperspende, Leichenbücher 1945–2016, LB 1945–1959, vgl. „Todesurteile österreichischen Volksgerichte 1945 bis 1948“, online unter: http://www.nachkriegsjustiz.at/prozesse/volksg/Tabelle_Todesurteile.php (25.06.2025).

42 AIA, Leichenverwaltung und Körperspende, Leichenbücher 1945–2016, LB 1945–1959.
